# Usage of brolucizumab as treatment for wet age-related macular degeneration (AMD) and polypoidal choroidal vasculopathy (PCV): A narrative review

**DOI:** 10.1097/MD.0000000000042666

**Published:** 2025-06-06

**Authors:** Siti Nursyazanie Jezani, Mae-Lynn Catherine Bastion

**Affiliations:** a Department of Ophthalmology, Hospital Canselor Tuanku Muhriz, Universiti Kebangsaan Malaysia, Kuala Lumpur, Wilayah Persekutuan, Malaysia.

**Keywords:** age-related macular degeneration, brolucizumab, polypoidal choroidal vasculopathy, vision loss

## Abstract

Intravitreal anti-vascular endothelial growth factor (anti-VEGF) is the gold standard treatment for neovascular age-related macular degeneration (nAMD) which is responsible for central vision loss. This results in loss of quality of life, comparably severe to having coronary artery disease or cancer. New anti-VEGF agents need to address issues of potency and durability as existing agents tend to lose their effect after 1 month thus, requiring multiple injections at close intervals. Brolucizumab is one of the latest anti-VEGF agents clinically proven to treat nAMD after on-label agents namely, ranibizumab and aflibercept. Several clinical trials were conducted on Brolucizumab to ensure its safety and efficiency before it is approved to be used as treatment. Brolucizumab maintains and improves retinal edema in nAMD leading to improved vision with longer intervals possible between injections. However, it also has a risk of intraocular inflammation. This review summarizes the evidence for brolucizumab in the treatment of nAMD.

## 1. Introduction

Age-related macular degeneration (AMD) affects the macula which is located at the center of the retina in the rear of the eye.^[1]^ This disease leads to progressive and irreversible loss of central vision^[2]^ which has become the most common cause of blindness among the elderly.^[3]^ Elderly, defined as persons aged 60 years and above is most prone to this eye disease.^[4,5]^ AMD causes blindness mostly in developed countries however, an increase in prevalence is seen in the East within the Asian population, particularly the polypoidal choroidal vasculopathy (PCV) variant.^[6]^ In the same study, contrary to Asians, the Western population’s predominant AMD subtype is choroidal neovascularization (CNV). A study by Wong et al^[7]^ shows a higher prevalence of AMD at 12.3% in Europeans aged 30 to 97 years old compared to Asians at 7.4% in the same age group. After glaucoma and cataract, AMD is the third leading cause of blindness worldwide in the elderly.^[2,3,8]^ In East Asia, visual impairment attributed to AMD has increased from only 5% in 1990 to 6.9% in 2010.^[9]^

AMD can be categorized into 2 types: “dry” or non-exudative and “wet” otherwise known as exudative or neovascular AMD (nAMD). The dry form is more common accounting for at least 80% of all AMD cases^[10]^ and is often asymptomatic.^[11]^ A person can also have a mixture of both dry and wet form which leads to irreversible central vision loss either partially or completely.^[12]^ This blurring of vision may be preceded by metamorphopsia, a visual distortion caused by retinal swelling which can be detected on an Amsler grid.^[13]^

Although nAMD only accounts for about 10% of AMD, it is responsible for 90% of blindness due to the disease.^[12,14]^ Individuals that initially had dry AMD might progress to nAMD with a probability of 10% to 20%.^[10]^ According to Liberski et al,^[14]^ nAMD is caused by abnormal growth of choroidal vessels under the macula called CNV. This CNV results in hemorrhage and fluid leakage which leads to fibrosis and permanent central vision loss.^[14]^ The fluid leakage and bleeding tend to be more extensive in the PCV subtype leading to widespread exudation thus, causing a larger field of vision loss.

According to global burden of disease (GBD 2019 blindness and vision impairment collaborators, vision loss expert group of the global burden of disease study^[15]^), 2 leading causes of vision impairment that occur due to aging are glaucoma and AMD. In 2002, AMD affected about 8.7% of the world population^[2,7,16]^ which is projected to increase yearly. Based on the systematic review by Wong et al,^[7]^ AMD is projected to affect 196 million of the world population in 2020 which can rise to 288 million in 2040. In a study by Christoforidis et al,^[17]^ AMD affects eyes asymmetrically. While AMD might be advanced in 1 eye, the other eye can be normal. Contradicting this statement is a study by Maguire et al^[18]^ which shows an advanced nAMD in 1 eye has a 42% risk of affecting the other healthy eye after 5 years of uncontrolled systemic hypertension. Hence, this condition like open angle glaucoma, may lead to bilateral blindness in an elderly person.

Based on a study by Freund et al,^[19]^ subtypes of AMD are classified based on the origin and location of CNV which can be categorized into 3 subtypes.^[20,21]^ Subtype 1 is known as PCV,^[22]^ subtype 2 is classic neovascularization^[20]^ whereas subtype 3 is retinal angiomatous proliferation.^[21]^

A study by Chakraborty et al^[23]^ in PCV AMD shows that both switch therapy (ST) and treatment-naïve (TN) eyes have positive responses to brolucizumab as it helps in the drying of fluids and improving vision. For subtype 2, REBA study shows a positive functional and anatomical response to brolucizumab with improving vision and central retinal thickness (CRT) in TN and ST patients.^[24]^ A case report for subtype 3 demonstrated the effectiveness of brolucizumab in visual gain and anatomical response even with a single dose injection.^[25]^

## 2. Indication for treatment of nAMD patients

### 2.1. Visual acuity and best corrected VA

According to Ackland et al^[26]^ based on data from 2015, approximately 3.44% of the world’s population which amounts to 253 million people, are visually impaired. From this group, 36 million (0.49%) are blind and 217 million (2.95%) are considered to have moderate to severe visual impairment (MSVI).^[26]^ Because visual impairment affects the psychological, functional, physical, social and emotional well-being of a person,^[27,28]^ this causes an individual’s quality of life to be drastically impacted.^[29]^

It is well known that nAMD results in central vision loss and decreased visual acuity (VA).^[30]^ For MSVI, AMD is the third commonest cause with 3.9% reported globally. An average loss of VA of about 3 lines (15 letters) in 2 years is caused by nAMD.^[31]^ A significant number of elderly individuals only start to seek treatment when both their eyes become affected. This is due to them being unaware of the visual loss in the initially affected eye until the other eye developed symptoms. Typically, the first eye will have developed disciform scarring by this time which is the end stage of AMD^[32]^ and patients may already have permanent loss of vision. Hence, the primary goal of treatment is to restore central vision for these patients.

Before anti-vascular endothelial growth factor (anti-VEGF) was prescribed, photodynamic therapy (PDT) was the only treatment option.^[33,34]^ Although it is less invasive than anti-VEGF by virtue of its administration via a peripheral vein such as the arm vein, a 2-year study by Brown et al^[35]^ found that PDT was only able to improve vision in 6.3% of patients, stabilize vision in 65.7% of patients while unable to save vision in the remainder. By contrast, ranibizumab which is an anti-VEGF, has much better VA outcomes than PDT with 3441% vision improvement and 89.9% to 90.0% stabilized vision.^[35]^ In addition, severe vision loss (≥30 letters lost from baseline) is reported in 16.1% of patients treated with PDT while only 1.4% was reported for groups receiving ranibizumab.

### 2.2. Usage of optical coherence tomography for diagnosis and monitoring of nAMD

Optical coherence tomography (OCT) is an imaging technology which noninvasively provides a high resolution detailed ocular structure image. It has been an essential tool for ophthalmology in the past 2 decades.^[36]^ This technology quantifies and visualizes retinal layers which help in the monitoring and diagnosis of retinal disease by way of macular imaging and analyzing the changes in morphology for disease monitoring. OCT is the gold standard diagnostic tool for AMD and is used to monitor disease progression from dry form to advanced.^[37]^ The common features of AMD include retinal thickness, abnormalities in choroidal vessels and development of geographic atrophy (GA). Intraretinal fluid (IRF) and subretinal fluid (SRF) compartments are markers of activity in the choroidal neovascular membrane in nAMD. CNV can also be visualized as a thickening in the Bruch membrane and by the presence of pigment epithelial detachment (PED).^[38]^ The OCT visualizes both the outer and inner segment of the eye. Valuable prognostic information is provided based on the retention of the photoreceptor cells comprising the inner segment.

## 3. Methods

The Web of Science database was used to search for peer-reviewed articles on AMD and brolucizumab from the date of database inception to March 2024. The literature search was performed using various combinations of keywords including “treatment,” “usage,” “AMD,” “nAMD,” “PCV,” “brolucizumab,” and “vision loss.” Inclusion criteria were as follows: articles in English, articles with full-text access and original research articles related to AMD. Eventually, 101 papers were included and cited.

## 4. Anti-VEGF agents

Before brolucizumab was introduced, several anti-VEGF have been approved to treat wet AMD which are pegaptanib (2004), ranibizumab (2006) and aflibercept (2011).^[39,40]^ Pegaptanib is rarely used now due to the superiority of the other factors. Bevacizumab (2005) is another effective agent which is used off-label. After brolucizumab, faricimab was introduced as the newest anti-VEGF.

### 4.1. Pegaptanib

Pegaptanib was the first pharmacological treatment used for nAMD.^[40]^ It has helped maintain VA in about 5% of patients^[41]^ but resulted in the inflammation of the anterior chamber, vitreous opacities and vitreous floaters.^[42]^ Approximately 1% of patients reportedly developed endophthalmitis from pegaptanib injections and a similar number developed traumatic cataract and retinal detachment as adverse effects.^[43]^ Pegaptanib modest nAMD treatment results led to a decline in its usage in favor of other more effective and safer agents. This 40 kilodalton (kDa) RNA aptamer binds with VEGF165 and is not used as anticancer agents.^[44]^ Its intravitreal half-life is 10 (±4) days for a 3 mg dose^[45]^ with the optimum dose of 0.3 mg/0.05 mL.^[46]^

### 4.2. Bevacizumab

Bevacizumab, an anticancer drug for breast and colorectal cancers,^[47]^ was discovered in 2005 to be effective for treatment of nAMD in CATT study but has remained off-label till now. In the CATT study, using the same drug administration schedule, bevacizumab and ranibizumab provide the same effectiveness on VA. However, bevacizumab was reported to have higher cases of serious adverse events compared to ranibizumab. Even though bevacizumab costs 40 times cheaper than ranibizumab and aflibercept, it requires repackaging into syringes in smaller doses for intraocular injection^[48]^ leading to higher risks of endophthalmitis.^[49]^ Its 149 kDa humanized IgG1 molecule that binds with VEGF-A in all isoforms^[50]^ helps in managing the disease. Its human intravitreal half-life in aqueous humor was 9.82 days^[51]^ with a typically given dose of 1.25 mg/0.05 mL.^[47,52]^

### 4.3. Ranibizumab

Ranibizumab is a 48 kDa molecule^[53]^ that binds with all isoforms of VEGF-A with its humanized antigen-binding fragment (Fab).^[41]^ Its half-life in aqueous humor with optimum dosage of 0.5 mg/0.05 mL is slightly shorter than bevacizumab at 7.19 days.^[54]^ In the landmark MARINA trial, ranibizumab prevents vision loss and improves VA in 24.8% and 33.8% of patients in doses of 0.3 mg and 0.5 mg respectively. Five out of 716 patients (1%) identified with presumed endophthalmitis and about 1.3% faced serious uveitis in this trial.^[31]^ In the landmark ANCHOR study, VA improved in 35.7% of patients in the 0.3 mg dosage group and 40.3% patients in the 0.5 mg dosage of ranibizumab.^[55]^ The trial also reported endophthalmitis and serious uveitis in 1% and 0.7% of patients respectively. Both trials showed significant improvements in VA in CNV patients taking ranibizumab injections with 0.5 mg being the optimum dosage for nAMD.^[41]^

### 4.4. Aflibercept

Aflibercept recombinant fusion protein binds with placental growth factor (plGF) and all isoforms of VEGF-A and VEGF-B.^[50]^ It has a molecular size of 115 kDa with an optimum dosage of 2.0 mg/0.05 mL and a half-life of 7.13 days in human eyes.^[56]^ A randomized controlled trial by Heier et al^[57]^ shows that no serious ocular adverse event was reported and that bimonthly injections after a 3-monthly initial loading dose are as effective as monthly ranibizumab injections. Aflibercept gives significant visual impact to patients that are resistant to ranibizumab and/or bevacizumab, with short-term visual improvement and reduction of CRT over 1 year of follow-up sessions.^[58]^ The EPIC trial by Kokame et al^[59]^ shows that PCV are responsive to aflibercept injections. Aflibercept stabilizes vision with significant improvements in terms of PED and resistant polyps. The hemorrhagic and exudative complications from polyps regress with aflibercept injections which is supported by a study by Choo et al^[60]^ Based on the 1 year study by Choo et al,^[60]^ this anti-VEGF works better in TN patients than ST patients, with a regression in persistent SRF.

### 4.5. Brolucizumab

Brolucizumab (RTH258) formerly known as ESBA1008, is a single-chain fragment variable (scFv) antibody that inhibits the binding process of ligand VEGF-A to vascular endothelial growth factor receptor-1 (VEGFR-1) and VEGFR-2.^[61]^ Brolucizumab was invented to be of a smaller molecular size at only 26 kDa^[62]^ to ensure that the drug can be delivered intraocularly in larger concentrations.^[24]^ With a smaller molecular size, it provides rapid systemic clearance that is essentially prerequisite to ensure lower chances of any systemic side effects.^[63]^ Brolucizumab also shows better potency as a drying agent based on clinical trials^[64]^ and was approved by the Food and Drug Administration (FDA) on October 8, 2019.^[65,66]^

### 4.6. Faricimab

Another player on the nAMD scene is faricimab which affects the angiopoietin-2 pathway in addition to VEGF-A blocking. Although it has completed Phase 3 trials, more studies are still needed to investigate its effectiveness compared to other available drugs.^[67]^ This drug is outside of the scope of this review.

### 4.7. Summary on usage of anti-VEGF prior to brolucizumab

A review of these clinical trials shows a 80% to 90% rate of response with anti-VEGF agents other than brolucizumab with a 30% significant improvement reported.^[68]^ This means at least 10% of individuals fail to respond to these therapies. These cases of nAMD would eventually lead to permanent scarring and loss of vision. Hence, a new agent to address the needs of this group was sought.

Other observations from a large series of nAMD patients including the need for frequent injections was seen in as many as 40% of patients. This was a reflection of the durability of the drugs with aflibercept demonstrating the ability to maintain retinal dryness with dosing of up to 6-month intervals in only 60.3% of patients.^[69]^ Hence, the search for agents with higher potency and durability. The dosing regimen most practiced in nAMD therapy is treat and extend (T and E). In the T and E dosing model, patients receive 3 months of loading dose given once per month followed by increasing intervals between doses determined by the presence or absence of fluid seen on OCT. In the absence of fluid while injection was given during the follow up, the next injection could be extended by a further 2 to 4 weeks. This allowed the disease to be better controlled while reducing the need for frequent visits to the doctor. This is in contrast to the pro re nata (PRN) or as needed regime in which patients return monthly and receive injections whenever fluid was seen to reappear. Another observation from dosing with these agents is the persistence of PED and SRF despite relatively dry retinas.

## 5. Clinical trials

### 5.1. Clinical trial evidence on brolucizumab for nAMD

The first-in-human trial for brolucizumab (RTH258) was conducted in Phase 1/2 of the SEE study (NCT01304693) to assess the safety, efficacy and tolerability of RTH258 in different dosages of single administration compared to 0.5 mg ranibizumab for nAMD treatment.^[70]^ One hundred and ninety-four TN patients with nAMD were included in this 6-month trial, with ages from 50 years and above. This study consists of 2 phases: Phase 1 on escalation phase dose to maximum feasible dose (MFD) of RTH258 (MFD = 6.0 mg), followed by Phase 2 on MFD expansion.

Phase 1 was randomized 5:2 with RTH258 (0.5, 3.0, or 4.5 mg) or 0.5 mg ranibizumab. After safety issues were reviewed, the ascending dosage was evaluated according to individual needs. Phase 2 was conducted in 2 parts in which patients were randomized 1:1 with 4.5 mg RTH258 or 0.5 mg ranibizumab in the first part. The second part enrolled patients randomized to 5:30:35:9 with either 0.5 mg, 3.0 mg, or 6.0 mg of RTH258 or 0.45 mg ranibizumab. This study concluded that 4.5 mg and 6.0 mg of RTH258 were non-inferior to 4.5 mg ranibizumab when assessed with CRT at month 1. In terms of safety issues, only mild adverse events were reported which resolved without any treatment after a few days. The development of RTH258 drugs was continued after this clinical trial. A 42-day OWL study (NCT01849692) conducted in TN nAMD patients concluded that the administration of RTH258 in microvolume (10 µL) by infusion or injection were efficacious in the majority of subjects to improve BCVA and CRT.^[71]^

Clinical trials continued in Phase 2 OSPREY study (NCT01796964) which was conducted to compare the safety and efficacy of brolucizumab with aflibercept for nAMD.^[72]^ Eighty-six TN nAMD patients were included and randomized 1:1 with 6 mg brolucizumab or 2 mg aflibercept. In this 56-week study, both groups received 3-monthly loading doses followed by treatment and assessment every 8 weeks (q8w) until week 40. For the brolucizumab group, the final q8w cycle was extended after week 40 to add another 2 cycles of every 12 weeks (q12w) treatment. This study concluded that at week 12 and 16, the mean BCVA from baseline for brolucizumab was non-inferior compared to aflibercept. During the q8w treatment, brolucizumab showed better stability on CRT even with unscheduled treatment compared to aflibercept. Fluid resolution in IRF and SRF in brolucizumab was also better with about 50% stable BCVA during the added cycles of q12w.

The first clinical trial to confirm the safety and effectiveness of brolucizumab for TN nAMD that included PCV before it was officially used to treat nAMD, was the Phase 3 HAWK and HARRIER trial by Dugel et al^[73]^ and the HAWK study by Ogura et al^[74]^ The aim of the HAWK and HARRIER study was to investigate whether injections of brolucizumab at every 8 or 12 weeks was as efficient as aflibercept in improving BCVA from baseline to 48 weeks. One thousand eight hundred and seventeen patients above 50 years old were selected from 408 sites with untreated and active CNV with SRF and/or IRF that alter central subfield thickness. Patients with BCVA of early treatment (ETDRS) between 23 and 78 (Snellen equivalent of 20/32 to 20/400) with no GA or fibrosis were included. One thousand and eighty-two of these patients were randomized 1:1:1 with aflibercept 2 mg, brolucizumab 3 mg or brolucizumab 6 mg in HAWK and 743 patients were randomized 1:1 with aflibercept 3 mg or brolucizumab 6 mg in HARRIER. BCVA changes from baseline for each brolucizumab group are non-inferior to aflibercept with more than 50% patients treated with 6 mg brolucizumab able to extend injection intervals to 12 weeks after the loading dose throughout 48 weeks. Brolucizumab also shows better resolution of fluid in IRF, SRF and subretinal pigment epithelium (sub-RPE) in week 16 with greater reduction in CRT from baseline to week 48.

### 5.2. Clinical trial evidence of brolucizumab for PCV

In the HAWK study for PCV, its primary objective is to compare the safety and efficacy of brolucizumab and aflibercept for PCV eyes in 96 weeks.^[74]^ From the previous HAWK study, 30 Japanese patients received 3 mg aflibercept and 39 patients are injected with 6 mg brolucizumab, all with a PCV diagnosis. With an 8-week interval dosage, brolucizumab had comparable BCVA with better fluid control and significant CRT reduction maintained to the 96th week. Of the 6 mg brolucizumab receivers, 76% are able to maintain a 12-week dosing interval throughout week 48 and 68% along week 96. The results of this trial found that brolucizumab was able to improve vision, manage fluids (SRF or IRF) and had better CRT reduction in patients with nAMD and PCV with administration intervals of up to 12 weeks.

The results for PCV described in the Everest study found that PDT with verteporfin monotherapy (71.4%) and PDT verteporfin combined with ranibizumab (78.0%) resulted in better regression of polyps in PCV patients compared to ranibizumab monotherapy (28.6%) at month 6.^[75]^ Nevertheless, 71.4% of PCV cases do not respond well or fully to anti-VEGF. This shows that PDT provides better outcome in PCV treatment. The study by Garweg et al^[76]^ found that brolucizumab could be loaded at 8-week intervals for 2 or 3 doses followed by the T&E or PRN regime based on the anatomical response of each patient.

More recently, trials involving brolucizumab have been performed for treatment resistant cases in comparison to other drugs known to have longer durability such as aflibercept. A study by Musiał-Kopiejka et al^[77]^ stated that brolucizumab provides more durability and effectiveness due to its smaller sized molecule and better tissue penetration capacity. With significant improvement in VA as well as functional and anatomical parameters, brolucizumab provides durability to extend the interval between injections for up to 12 weeks.

## 6. Real-world evidence for brolucizumab usage

Real-world evidence (RWE) for the usage of a new drug is invaluable to ensure its application in the real world is as effective as in landmark studies. This also allows clinicians to gauge the effectiveness of a drug in patients who do not fulfill the inclusion criteria of the studies. Other benefits include the ability to determine if other ethnic groups show the same results and if there are any safety issues.

Real-world evidence supports the findings that brolucizumab significantly improves vision in TN cases. As for ST cases and cases which have been less responsive to other anti-VEGF agents, a less significant improvement is seen.^[78]^ Nevertheless, the CRT significantly reduces in both ST and TN patients from these real-world reports.^[24,78]^

The safety issues associated with brolucizumab resulted in its usage to be mainly in recalcitrant nAMD cases in which fluid in the IRF, SRF, or PED compartments persist. It was found to be particularly effective in drying the retina and reducing PED heights. Real-world studies also recorded responses to brolucizumab in Caucasian and Asian populations. Reports from other regions including Asia have been slow. Nevertheless, a recent report from Japan found brolucizumab injections effective in improving BCVA and fluid resolution in a short-term period.^[79]^

Table 1 presents RWE data from previously conducted studies on brolucizumab injections for neovascular AMD (nAMD), reported between 2020 and 2023. The first study, BREW by Sharma et al^[80]^ in 2020 reported no adverse events. In contrast, incidence of (IOI) cases were reported by Maruko et al,^[81]^ Enríquez et al^[82]^ and Bullirsch et al^[83]^ Additionally, Bilgic et al^[24]^ noted a case of vascular occlusion following brolucizumab administration. Studies by Haensli et al^[84]^ and PROBE study by Bilgic et al^[85]^ indicated a favorable safety profile, with no adverse events recorded during the follow-up period. Conversely, BRAILLE study by Chakborty et al^[86]^ reported 2 cases of subretinal hemorrhage, and 1 patient in Montesel et al^[87]^ study developed IOI after injection. In 2022, Chakraborty et al^[23]^ reported no adverse event, while patients in the studies by Abdin et al^[88]^ and Giunta et al^[89]^ experienced IOI. Tanaka et al^[79]^ noted a case of iritis. In 2023, IOI was again noted in the studies by Coney et al^[90]^ and Yeom et al^[91]^ while REBA extension study by Bilgic et al^[78]^ reported 3 cases of vitritis. These findings highlight the variability in reported ocular adverse events across studies.

**Table 1:** Previous real-world evidence on brolucizumab for AMD.

Year published	Sample size	Country involved	Change in BCVA	Change in CRT	Fluid resolution	Adverse event	Reference
July 2020	42 eyes, 42 patients (ST)	USA	*P* = .33	*P* = .0027	IRF (19 eyes, 7 eyes resolved, 36.8%) SRF (38 eyes, 15 eyes resolved, 39.4%) PED (31 eyes, 2 eyes resolved, 6.4%)	No adverse event reported	BREW study Sharma et al^[80]^
March 2021	127 eyes, 127 patients 43 eyes (TN) 84 eyes (ST)	Japan	NA	NA	NA	12 IOI includes: 4 IOI with retinal vasculitis 2 IOI with retinal vasculitis and retinal vascular occlusion	Maruko et al^[81]^
April 2021	172 eyes, 152 patients 4 eyes (TN) 168 eyes (ST)	USA	*P* = .65	*P* = .003	IRF (19 eyes) SRF (77 eyes) IRF and SRF (17 eyes) First injection (50 eyes resolved, 44.2%) Second injection (6 eyes resolved, 5.3%)	14 IOI 1 endophthalmitis 1 IOI, uncertain association with brolucizumab 1 vitreous opacity without IOI 1 IOP elevation 1 PVD	Enríquez et al^[82]^
April 2021	63 eyes, 57 patients (ST)	Germany	*P* = .115	*P* < .001	IRF (27 eyes, 11 eyes resolved, 40.7%) SRF (42 eyes, 24 eyes resolved, 57.1%) PED (30 eyes, 6 eyes resolved, 20%)	7 IOI 1 retinal vasculitis	SHIFT study Bulirsch et al^[83]^
June 2021	105 eyes, 78 patients 80 eyes (TN) 25 eyes (ST)	India, Germany	*P* = .014	TN, *P* = .021 ST, *P* = .013	IRF (23 eyes TN, 57 eyes ST) SRF (12 eyes TN, 31 eyes ST) PED (2 eyes TN, 24 eyes ST) *No resolution data	1 vascular occlusion 1 macular hole	REBA study Bilgic et al^[24]^
June 2021	12 eyes, 12 patients (ST)	Switzerland	NA	NA	IRF (4 eyes, reduced in 2 eyes) SRF (3 eyes, reduced in 2 eyes, increased in 1 eye)	No adverse event reported	Haensli et al^[84]^
September 2021	27 eyes, 27 patients (TN)	India	*P* = .014	*P* = .013	IRF (18 eyes) SRF (8 eyes) PED (16 eyes) First injection (7 eyes resolved, 25.9%) Second injection (13 eyes resolved, 48.2%) Third and more injections (7 eyes resolve, 25.9%) *Recurrence in exudation (23 eyes, 85.2%)	No adverse event reported	PROBE Study Bilgic et al^[85]^
September 2021	94 eyes, 94 patients 20 eyes (TN) 74 eyes (ST)	India	*P* < .0001	*P* < .0001	IRF (84 eyes, 33 resolved, 39.29%) SRF (71 eyes, 11 eyes resolved, 15.5%) PED (21 eyes, 5 eyes resolved, 23.81%)	2 subretinal hemorrhages 1 RPE tear	BRAILLE Study Chakraborty et al^[86]^
November 2021	19 eyes, 19 patients 15 eyes (ST) 4 eyes (TN)	Switzerland	*P* = .778	*P* = .001	IRF (12 to 3 eyes, *P* = .065) SRF (17 to 3 eyes, *P* = .111) PED (16 to 14 eyes, *P* = .811)	1 IOI	Montesel et al^[87]^
April 2022	21 eyes, 21 patients 16 eyes (ST) 5 eyes (TN)	India	*P* = .01	*P* < .001	IRF (17 eyes, 10 resolved and 7 reduced) SRF (17 eyes, 8 resolved and 9 reduced) PED (21 eyes, 5 resolved and 16 reduced height)	NA	Chakraborty et al^[23]^
May 2022	21 eyes, 19 patients (ST)	Germany	*P* = .6	*P* = .01	IRF (57 to 57 eyes, *P* = .8) SRF (71 to 50 eyes, *P* = .03) PED (38 to 21 eyes, *P* = .01)	2 IOI	Abdin et al^[88]^
July 2022	58 eyes, 57 patients (TN)	Japan	*P* = .002	*P* < .0001	IRF and SRF: resolve 67% at month 1, 88% at month 2 and 91% at month 3. PED: 10 to 1 eye resolved, 8 eyes decrease in height at month 3.	8 iritis	Tanaka et al^[79]^
August 2022	73 eyes, 73 patients (ST)	Canada	*P* = .057	*P* = .0002	IRF (42 eyes, reduced by 66.6%) SRF (59 eyes, reduced by 62.7%)	3 IOI	Giunta et al^[89]^
February 2023	174 eyes, 154 patients (ST)	USA	*P* = .42	*P* < .0001	NA	21 IOI	Coney et al^[90]^
July 2023	81 eyes, 81 patients (ST)	South Korea	*P* = .006	*P* < .001	38% (31 eyes) dry macula after first injection >90% (76 eyes) reduced in first month	1 retinal vascular occlusion 7 IOI	Yeom et al^[91]^
December 2023	91 eyes, 67 patients 71 eyes (ST) 21 eyes (TN)	India, Germany	*P* = .001 (ST) *P* = .01 (TN)	*P* = .011 (ST) *P* = .018 (TN)	IRF (23 eyes TN, 57 eyes ST) SRF (12 eyes TN, 31 eyes ST) PED (2 eyes TN, 24 eyes ST) *No resolution data	Addition of: 3 vitritis cases from first analysis	REBA extension Study Bilgic et al^[78]^

BCVA = best corrected visual acuity, CRT = central retinal thickness, IOI = intraocular inflammation, IOP = intraocular pressure, IRF = intraretinal fluid, NA = not available, PED = retinal pigment epithelial detachment, PVD = posterior vitreous detachment, RPE = retinal pigment epithelium, SRF = subretinal fluid, ST = switch therapy, TN = treatment-naïve.

*P* < .05, statistically significant (have effect/changes).

## 7. Safety issues

Soon after its launch in 2019, brolucizumab was found to have significant issues in causing IOI which was previously not detected in the landmark trials before Phase 3 HAWK and HARRIER. A higher incidence of IOI was recorded in 6 mg brolucizumab compared to aflibercept at week 48 with HAWK (5.3% vs 0.3%) and HARRIER (2.7% vs 0.8%) in this clinical trial.^[92]^ The IOI related cases reported were from anterior chamber flare, anterior chamber cells, anterior chamber inflammation, eye inflammation, vitreous haze, chorioretinitis, uveitis, vitritis, iritis and iridocyclitis. The most reported cases are from uveitis and iritis. The MERLIN trial which was conducted after HAWK and HARRIER is not included in the discussion as the trial was terminated due to an even higher IOI incidence with an addition of retinal vascular occlusion and retinal vasculitis cases.^[93]^ This led to an FDA investigation after which brolucizumab had a safety label. Subsequently, brolucizumab RWE supported that there was an unusually high incidence of IOI.

The United States Prescribing Information announced that there was a 4% incidence rate of IOI seen with brolucizumab of which 2% overall developed retinal vasculitis and 1% had vascular occlusion. The IOI responded well to the established regime of corticosteroids which treated 86.8% to 87.1% of cases and permanent vision loss is not often seen.^[94,95]^ There were 75% of IOI cases found within the first 6 months of administration^[95]^ hence, patients had to be counseled on the risk of IOI prior to the administration of brolucizumab. Counselling included providing information on symptoms of IOI namely, conjunctival injection, floaters, blurred vision, ocular pain and scotoma.^[96,97]^ Females^[97]^ and those with known autoimmune disease^[98]^ are more at risk to develop IOI.

Adverse effects were seen to occur regularly in most of the series with large numbers of patients. After 4% of IOI adverse events were reported in the Dugel et al^[72]^ study, a post hoc analysis was conducted by Singer et al^[95]^ for safety measures and treatment management. With a median time of 18 days for IOI onset from the last brolucizumab administration, 5.7% of cases were reported as severe, 40.0% moderate and 54.3% were classified as mild. Approximately 70% of these IOI cases happened after 4 injections within 6 months of the first brolucizumab injection. Studies report that 87.1% of the IOI cases were treated with topical corticosteroids intraocularly and systemically. Out of 49 eyes, 79.6% completely resolved, 10.2% resolved with sequelae while 10.2% remain unresolved.^[95]^ A case report by Kurup et al^[99]^ shows that mild IOI caused by brolucizumab injections can be treated successfully with a single injection of 40 mg/mL posterior sub-tenon triamcinolone (PST) that helps in stabilizing and resolving the inflammation which improved vision. A Japanese study highlights that the female gender and those with previous inflammation history are particularly prone to IOI.^[97,100]^

## 8. Other applications of brolucizumab in the future

Brolucizumab is only FDA approved for AMD treatment. However, based on a review registered on PROSPERO (CRD42022382625), brolucizumab also has significant advantages in diabetic macular edema (DME) and DR.^[101]^ It has the potential to improve BCVA and CRT as well as lower the treatment burden in DME and DR patients. Nevertheless, this will not be discussed further as it is outside of the review topic.

## 9. Conclusion

Brolucizumab is a highly effective and durable drug for the management of nAMD. At the beginning of the study, this drug was innovated to lessen patients’ treatment burden apart from its effectiveness in controlling disease activity. However, the drug has significant safety issues which need to be addressed. This might limit its widespread application as a first-line agent.

## Author contributions

**Conceptualization:** Siti Nursyazanie Jezani, Mae-Lynn Catherine Bastion.

**Data curation:** Siti Nursyazanie Jezani, Mae-Lynn Catherine Bastion.

**Formal analysis:** Mae-Lynn Catherine Bastion.

**Funding acquisition:** Mae-Lynn Catherine Bastion.

**Investigation:** Siti Nursyazanie Jezani.

**Supervision:** Mae-Lynn Catherine Bastion.

**Validation:** Mae-Lynn Catherine Bastion.

**Visualization:** Siti Nursyazanie Jezani.

**Writing – original draft:** Siti Nursyazanie Jezani.

**Writing – review & editing:** Siti Nursyazanie Jezani, Mae-Lynn Catherine Bastion.
